# Sun protection and skin cancer screening after childhood cancer—A report from the Swiss Childhood Cancer Survivor Study

**DOI:** 10.1002/cncr.70364

**Published:** 2026-03-29

**Authors:** Carina Nigg, Maša Žarković, Philippa Jörger, Eva Maria E. Tinner, Calogero Mazzara, Eva Brack, Paul Castle, Alexander Navarini, Christina Schindera, Claudia E. Kuehni

**Affiliations:** ^1^ Childhood Cancer Research Group Institute of Social and Preventive Medicine University of Bern Bern Switzerland; ^2^ Graduate School for Health Sciences University of Bern Bern Switzerland; ^3^ Division of Pediatric Hematology and Oncology Inselspital Bern University Hospital University of Bern Bern Switzerland; ^4^ University Institute of Internal Medicine Cantonal Hospital Baselland Liestal Switzerland; ^5^ Medical Faculty University of Basel Basel Switzerland; ^6^ Pediatric Hematology‐Oncology Unit Division of Pediatrics Department “Woman‐Mother‐Child” University Hospital and Lausanne University Lausanne Switzerland; ^7^ Patient Representative Bottmingen Switzerland; ^8^ Department of Dermatology University Hospital Basel Basel Switzerland; ^9^ Department of Oncology/Haematology University Children’s Hospital Basel UKBB Basel Switzerland

**Keywords:** dermatologic examination, dermatology, follow‐up studies, health behavior, pediatric oncology, skin cancer surveillance, skin neoplasms, survivorship

## Abstract

**Background:**

Childhood cancer survivors (CCS) face elevated skin cancer risk, especially after radiotherapy or hematopoietic stem cell transplantation (HSCT). The authors evaluated the prevalence and predictors of sun protection, sunburn, and physician skin examination (PSE) among CCS in Switzerland.

**Methods:**

The authors surveyed CCS diagnosed <21 years and surviving ≥5 years after diagnosis about sun protection, sunburns during last summer, and PSE within the last year. They retrieved cancer‐related data from the Swiss Childhood Cancer Registry and used multivariable logistic regression, stratified by age group, to identify predictors.

**Results:**

The authors included 1048 children (5–15 years), 572 adolescents (16–19 years), and 1959 adults (≥20 years). Regular sun protection was reported by 89% of children, 65% of adolescents, and 77% of adults, and sunburns by 23%, 49%, and 43%, respectively. PSE prevalence among those treated with radiotherapy was 21%, 18%, and 17%, and among HSCT recipients 36%, 28%, and 28%, respectively. Radiotherapy was unrelated to sun protection and PSE, but associated with fewer sunburns (odds ratio [OR], 0.63–0.77). HSCT recipients were more likely to have attended a PSE (OR, 2.06–3.75), but not radiotherapy recipients. Across age groups, survivors born more recently were less likely to protect from sun (OR, 0.94–0.97) and more likely to report sunburn (OR, 1.04–1.14).

**Conclusion:**

Sunburn prevalence was high despite good sun protection. Only few at heightened risk for skin cancer due to their treatment history attend PSEs as recommended by the Children’s Oncology Group. Health care practitioners should systematically integrate yearly PSE after radiotherapy or HSCT and encourage consistent sun protection, particularly among younger generations and adolescents.

**Trial Registration:**

Clinicaltrials.gov (NCT03297034).

## Abstract 2

One in three childhood cancer survivors experienced a sunburn the previous summer, and only one in five childhood cancer survivors treated with radiotherapy or HSCT meets current recommendations for skin cancer screening. This highlights the need to systematically integrate sun safety education and skin cancer screening into follow‐up care.

## INTRODUCTION

Melanoma, the most serious form of skin cancer, has increased markedly in Switzerland over the past 40 years: registered cases rose from approximately 4000 (1982–1986) to 16,500 (2017–2021).^1^ It is now the fifth most common cancer in Switzerland,^2^ placing the country among those with the highest melanoma incidence in Europe.^3^ Major risk factors include genetic predisposition (particularly fair, sun‐sensitive skin) and ultraviolet radiation exposure.^4^ Among childhood cancer survivors (CCS), cancer treatments such as radiotherapy, certain chemotherapies (e.g., alkylating agents or bleomycin), and hematopoietic stem cell transplantation (HSCT) increase the risk of basal and squamous cell carcinomas^5^, ^6^ and melanomas.^7^ Radiotherapy can damage epithelial and vascular endothelial tissues, leading to persistent oxidative stress and chronic inflammation that impair long‐term skin repair.^8^ Neurotoxic chemotherapies may cause lasting alterations in cutaneous sensory nerve function, potentially affecting skin sensitivity to ultraviolet exposure.^9^ CCS are twice as likely to develop melanomas compared to their peers without cancer history^10^ and have a 30‐fold higher risk of developing basal cell carcinoma already in young adulthood.

The Children’s Oncology Group (COG) recommends annual physician skin examination (PSE) for survivors treated with radiation or HSCT and sun protection for all CCS^11^ to reduce skin cancer mortality.^12^, ^13^, ^14^ Yet, we know little about sun protection and PSE after childhood cancer. Existing studies focus primarily on the United States,^15^, ^16^, ^17^, ^18^, ^19^ with different sun protection practices compared to Europe,^20^ on adult CCS,^15^, ^16^, ^17^, ^21^, ^22^ or rely on data collected 15–20 years ago.^15^, ^16^, ^19^, ^21^ We aim to describe sun protection, sunburn, and PSE in participants of the Swiss Childhood Cancer Survivor Study (SCCSS), a population‐based study of pediatric, adolescent, and adult CCS with data from new survivors having been collected repeatedly 2007–2022. In particular, this study evaluates prevalence and predictors of sun protection, sunburn, and PSE, and assesses compliance with PSE as recommended by the COG.

## MATERIALS AND METHODS

### Study design and study population

The Swiss Childhood Cancer Survivor Study (SCCSS) is a population‐based study nested in the Childhood Cancer Registry (ChCR). Detailed methods of the SCCSS are available elsewhere.^23^ The ChCR includes all children and adolescents diagnosed with leukemias, lymphomas, central nervous system (CNS) tumors, malignant solid tumors, or Langerhans cell histiocytosis before age of 20 years in Switzerland since 1976.^24^ In the SCCSS, we sent questionnaires to all CCS who had survived ≥5 years since their initial diagnosis. Because some survivors were diagnosed at birth or early infancy, this eligibility criterion includes children as young as 5 years at the time of the study. For CCS 5–15 years old at the time of the study, parents filled in the questionnaire. Questionnaires are available in German, French, and Italian. Survivors of this study participated in the SCCSS 2007–2022. Ethical approval was granted by the ethics committee of the canton of Bern, Switzerland (KEK‐BE: 166/2014 and 2021‐01462).

### Sun protection, sunburn, and physician skin examination

We derived all questions on sun protection, sunburn, and physician skin examination from the Swiss Health Survey, a nationally representative health survey among Swiss residents ≥15 years old.^25^

#### Sun protection

We asked all participants how consistently they protect themselves from the sun (e.g., using sunscreen, seeking shade, or wearing protective clothing). Response options ranged from “Always” to “(Almost) never” (Table S1). For risk factor analysis, we defined good sun protection as protecting always or mostly from sun, and poor sun protection as protecting rarely or never from sun. Self‐reported sun protection has been shown to be adequate for reporting sunscreen use among children and adults, and for parental proxy‐reporting on sunscreen use.^26^ Agreement between self‐ and proxy‐reported sunscreen use, sunburns the past summer, and skin color is good between early adolescents and parents.^27^

#### Sunburn

We asked all participants whether they had experienced one or more sunburns in the past summer (response options no/yes).

#### Physician skin examination

We asked all participants whether they had ever had their skin or moles examined by a physician within the last 12 months, more than 12 months ago, or never. We defined compliance with the COG guidelines as having had the skin examined within the last 12 months, and no compliance if they had never had a PSE or if it was more than 12 months ago. Validity of self‐reported skin examinations has been reported good to excellent.^28^

### Explanatory variables

#### Sociodemographic characteristics

We assessed the following sociodemographic characteristics via questionnaires: Swiss language region, parental and participant nationality, and parental education. We categorized language region into German and French/Italian. For migration background and parental education, we applied the definitions of the Federal Statistics Office.^29^, ^30^ If both parents were born Swiss or if the participant and at least one parent were born Swiss, we defined this as no migration background.^29^ For those with migration background, we applied the United Nations’ geographic coding scheme to obtain some information on their cultural background.^31^ For educational background, we considered the highest education level achieved by either parent as the primary education indicator across all age groups, including adult survivors, to ensure comparability across age groups, since several young adult survivors were still in education, and because parental education continues to influence health behaviors in adulthood.^32^, ^33^ We divided education into three categories: primary (compulsory schooling only), secondary (higher schooling or vocational training), or tertiary (upper vocational education or university or technical college).^30^

#### Cancer‐related characteristics

We received the following cancer‐related characteristics from the ChCR: sex, date of birth, age at diagnosis, and diagnosis according to the International Classification of Childhood Cancer, third edition (ICCC‐3).^34^ Treatment information included chemotherapy, radiotherapy, HSCT, and relapse (all no/yes).

#### Skin type

We used the Fitzpatrick scale to assess skin phototyping. This six‐level system classifies skin type based on genetic predisposition, skin reaction to sun exposure, and tanning ability.^35^

### Statistical analysis

We stratified analyses by age group: children (5–15 years), adolescents (16–19 years), and adults (>20 years). We included all participants in the analysis who responded to at least one of the questions in the questionnaire section about sun protection and PSE.^11^ We calculated the number and proportion of CCS for each of the response options for sun protection, sunburn, and PSE. To assess compliance with COG recommendations, we further assessed number and proportion of CCS treated with radiotherapy or HSCT who attended a PSE within the last 12 months.^11^ To investigate factors associated with sun protection, sunburn, and PSE, we applied multivariable logistic regression analysis. We decided a priori to include age at study, sex, migration background, language region, parental education, radiotherapy, and HSCT as exposures in all models, and also considered year of study and birth year to account for potential age–period–cohort effects. Because age, year of study, and birth year are perfectly collinear, we compared models including these predictors separately and in combination.^36^ Model fit based on Akaike (AIC) and Bayesian (BIC) information criteria indicated that including all three was unnecessary. Models including birth year consistently showed the best fit when also considering all other exposures (Table S2) and were thus used in final analyses, whereas we retained models with year of study for sensitivity analyses. Because we had only asked a subsample of study participants about skin type, we performed a sensitivity analyses including this exposure. Additional sensitivity analyses included the meteorological season in which the questionnaire was filled in as exposure, and for adult survivors, their own highest education they had obtained at the time of study besides their parent’s education. Given that several outcomes were common, we also conducted Poisson regression with robust error variances to estimate the relative risk (RR) and enable direct comparison between exposure groups.^37^

## RESULTS

In total, of 5940 eligible survivors, 5427 were contacted. Of those, 3768 (69%) filled in the questionnaire and 3579 (66%) responded to at least one question regarding sun exposure or PSE (1048 pediatric, 572 adolescent, and 1959 adult CCS) (Figure S1). Median age at study was 21 years (interquartile range [IQR], 15–27), median age at diagnosis 7 years (IQR, 3–13), and median time since diagnosis 12 years (IQR, 8–19). Leukemia was the most common cancer diagnosis (32%), followed by lymphomas (17%) and CNS tumors (16%). Overall, 77% had received chemotherapy, 31% radiotherapy, and 7% HSCT (Table 1).

**TABLE 1:** Characteristics of childhood cancer survivors included in sun exposure and physician skin examination study.

	Children (5–15 years), *N* = 1048	Adolescents (16–19 years), *N* = 572	Adults (≥20 years), *N* = 1959
Socio‐demographic characteristics			
Age at time of questionnaire, median [IQR]	12 [10–14]	18 [17–19]	27 [23–33]
Year of birth [IQR]	2003 [1999–2007]	1992 [1991–1994]	1982 [1976–1988]
Year of study participation, No. (%)			
2007–2013^a^	450 (43)		
2007–2009		257 (45)	1,076 (55)
2010–2013		179 (31)	413 (21)
2015–2017	314 (30)	73 (13)	232 (12)
2021–2022	284 (27)	63 (11)	238 (12)
Female, No. (%)	464 (44)	273 (48)	937 (48)
Migration background, No. (%)	229 (22)	119 (21)	341 (17)
UN geographic region, No. (%)^b^			
Northern/Western/Eastern Europe	835 (80)	465 (82)	1590 (83)
Southern Europe	118 (11)	79 (14)	207 (11)
Rest of the world	45 (4)	23 (4)	20 (1)
Language, No. (%)			
German	728 (70)	399 (70)	1382 (71)
French	276 (26)	155 (27)	516 (26)
Italian	44 (4)	18 (3)	61 (3)
Parental highest education, No. (%)^b^			
Primary education	81 (8)	33 (6)	193 (10)
Secondary education	361 (34)	344 (60)	1001 (51)
Tertiary education	577 (55)	171 (30)	657 (34)
Cancer‐related characteristics			
ICCC3 main group, No. (%)			
Leukemias	417 (40)	188 (33)	548 (28)
Lymphomas	66 (6)	84 (15)	466 (24)
CNS tumors	169 (16)	104 (18)	291 (15)
Neuroblastoma	93 (9)	31 (5)	52 (3)
Retinoblastoma	61 (6)	14 (2)	28 (1)
Renal tumor	84 (8)	44 (8)	71 (4)
Hepatic tumor	15 (1)	3 (<1)	12 (<1)
Malignant bone tumors	18 (2)	19 (3)	117 (6)
Soft tissue sarcomas	65 (6)	36 (6)	124 (6)
Germ cell tumors	23 (2)	15 (3)	127 (7)
Other	4 (<1)	8 (1)	68 (4)
Langerhans cell histiocytosis	33 (3)	26 (5)	55 (3)
Age at diagnosis, median [IQR]	3 [2–5]	6 [3–10]	12 [6–16]
Time since diagnosis, median [IQR]	8 [7–10]	11 [8–15]	18 [11–23]
Year of diagnosis, median [IQR]	2010 [2003–2011]	2000 [1995–2004]	1990 [1985–2000]
Radiotherapy, No. (%)	197 (19)	164 (29)	749 (38)
Chemotherapy, No. (%)	859 (82)	454 (79)	1456 (74)
HSCT, No. (%)	82 (8)	38 (7)	115 (6)
Relapse, No. (%)	37 (4)	15 (3)	55 (3)

Abbreviations: CNS, central nervous system; HSCT, hematopoietic stem cell transplantation; ICCC3, International Classification of Childhood Cancer, 3rd edition; IQR, interquartile range; UN, United Nations.

^a^ Because children were gradually included in the study, there were only few study participants before 2010, so we summarized the years 2007–2013 for children.

^b^ Percentages are based on the available *N*, not adding up to 100%.

### Prevalence of sun protection, sunburn, and PSE

**FIGURE 1:**
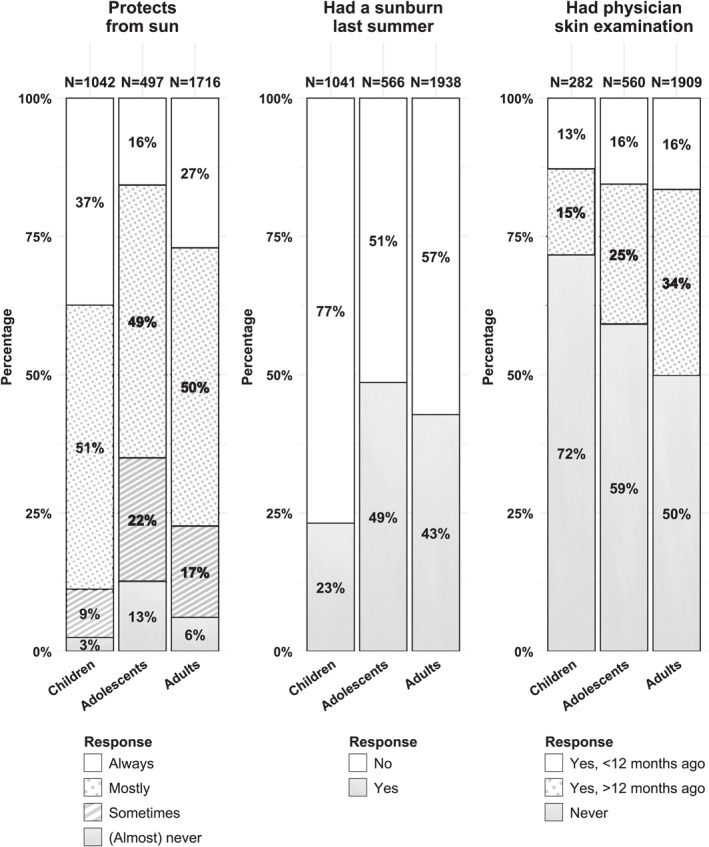
Prevalence of sun protection, sunburn, and physician skin examinations among childhood cancer survivors stratified by age group.

Overall, 79% of survivors reported good sun protection (Table S3). Sun protection differed by age group (Figure 1): 88% of children, 65% of adolescents, and 77% of adults reported protecting always or mostly from sun. Nonetheless, 38% reported at least one sunburn the previous summer, with the highest prevalence among adolescents (49%).

Because of the high prevalence of sunburn despite good sun protection, we compared survivors who reported good sun protection and did not experience sunburn with those who reported good sun protection but experienced sunburn. Among children and adults, survivors who reported sunburn despite good sun protection were more recently born (indicating a birth cohort rather than age effect), had participated more recently in the study, and had been diagnosed more recently. Children (25%) and adults (44%) without migration background had a higher proportion of reporting sunburn despite protection compared to those with migration background (children: 18%; adults: 26%). Across all age groups, the proportion reporting sunburn despite good sun protection was lower among survivors treated with radiotherapy (17%–37%) compared to those without (25%–52%) (see Table S4).

**FIGURE 2:**
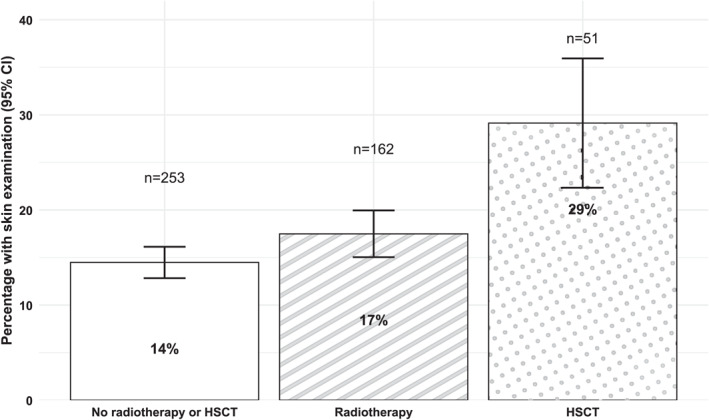
Compliance with physician skin examination attendance within the last 12 months as recommended by the Children’s Oncology Group for childhood cancer survivors treated with radiotherapy or hematopoietic stem cell transplantation. The *n* refers to the number of survivors who attended a physician skin examination within the last 12 months. HSCT, hematopoietic stem cell transplantation.

PSE compliance with the COG recommendation was low (Figure 2; Table S5). Among survivors treated with radiotherapy, 17% had a PSE within the past 12 months compared with 15% of those without radiotherapy. Among HSCT survivors, 29% reported a recent PSE versus 15% without HSCT. These patterns were similar across age groups.

### Factors associated with sun protection and sunburn

Table 2 shows results from logistic regression analyses, Table S6 results from Poisson regression. Across age groups, good sun protection was less likely among survivors with a migration background compared to those without, whose parents had only primary education compared to tertiary education, and among survivors born more recently, indicating a birth cohort effect. Independent of birth cohort, among children, higher age was associated with less sun protection (odds ratio [OR], 0.78; 95% CI, 0.71–0.85). Male adolescent (OR, 0.37; 95% CI, 0.24–0.56) and adult survivors (OR, 0.51; 95% CI, 0.40–0.65) were less likely to protect from sun than females. Radiotherapy and HSCT treatment were unrelated to sun protection.

**TABLE 2:** Factors associated with sun protection, sunburn, and physician skin examinations in multivariable logistic regression in child, adolescents, and adults CCS.

	Children (5–15 years)			Adolescents (16–19 years)				Adults (≥20 years)		
	OR	95% CI	*p*	OR		95% CI	*p*	OR	95% CI	*p*
Sun protection	*N* = 1013			*N* = 476				*N* = 1626		
Age at study, years	0.73	0.66–0.82	<.001	0.91	0.77–1.08		.294	1.01	0.98–1.04	.543
Male^a^	0.72	0.47–1.09	.122	0.38	0.25–0.57		<.001	0.51	0.40–0.65	<.001
Migration background^b^	0.64	0.39–1.07	.083	0.60	0.35–1.01		.053	0.58	0.43–0.79	.001
French/Italian language region^c^	1.25	0.80–2.00	.331	1.05	0.67–1.66		.822	1.01	0.77–1.32	.967
Parental education^d^										
Secondary	1.98	0.98–3.93	.053	1.34	0.58–3.11		.489	1.30	0.87–1.94	.193
Tertiary	1.98	0.99–3.84	.048	1.92	0.79–4.65		.149	1.55	1.01–2.36	.042
Radiotherapy^e^	1.34	0.78–2.45	.310	1.58	1.01–2.52		.050	1.20	0.93–1.56	.155
HSCT^f^	1.09	0.50–2.75	.842	1.59	0.67–4.11		.314	1.01	0.60–1.77	.962
Birth year	0.94	0.89–0.99	.027	0.95	0.91–1.00		.047	0.97	0.94–0.99	.004
Sunburn	*N* = 1013			*N* = 542				*N* = 1837		
Age at study, years	1.28	1.18–1.38	<.001	1.03	0.89–1.18		.719	1.01	0.99–1.03	.411
Male^a^	0.98	0.73–1.33	.916	0.73	0.52–1.03		.076	1.05	0.86–1.27	.632
Migration background^b^	0.53	0.34–0.81	.004	0.72	0.44–1.17		.187	0.48	0.36–0.63	<.001
French/Italian language region^c^	0.95	0.68–1.31	.740	1.11	0.75–1.63		.605	0.85	0.68–1.05	.137
Parental education^d^										
Secondary	1.09	0.58–2.13	.801	1.08	0.48–2.46		.853	1.18	0.82–1.70	.379
Tertiary	0.90	0.49–1.76	.759	1.33	0.58–3.12		.501	1.47	1.01–2.14	.044
Radiotherapy^e^	0.67	0.43–1.01	.065	0.63	0.43–0.93		.022	0.77	0.63–0.94	.012
HSCT^f^	0.61	0.30–1.14	.143	1.45	0.72–2.95		.303	0.62	0.39–0.95	.031
Birth year	1.14	1.10–1.19	<.001	1.04	0.99–1.08		.101	1.07	1.05–1.09	<.001
Physician skin examination	*N* = 275			*N* = 538				*N* = 1807		
Age at study, years	0.27	0.07–0.94	.044	1.02	0.83–1.24		.874	1.04	1.01–1.07	.019
Male^a^	0.97	0.45–2.10	.942	1.38	0.85–2.27		.194	0.65	0.50–0.83	.001
Migration background^b^	0.87	0.33–2.08	.757	1.28	0.67–2.36		.435	1.35	0.95–1.89	.086
French/Italian language region^c^	2.21	0.99–4.93	.051	1.08	0.63–1.81		.773	1.39	1.06–1.82	.016
Parental education^g^										
Secondary				1.55	0.51–5.80		.471	0.96	0.62–1.50	.844
Tertiary	3.27	1.20–10.79	.032	1.58	0.51–6.09		.460	1.28	0.82–2.02	.289
Radiotherapy^e^	2.62	0.94–6.86	.054	1.15	0.67–1.93		.596	1.01	0.77–1.32	.935
HSCT^f^	3.75	1.39–9.83	.008	2.06	0.87–4.55		.083	2.07	1.29–3.27	.002
Birth year	0.31	0.08–1.09	.072	1.00	0.94–1.06		.927	1.01	0.99–1.04	.339

Abbreviations: CCS, childhood cancer survivors; HSCT, hematopoietic stem cell transplantation; OR, odds ratio.

^a^ Reference: female.

^b^ Reference: no migration background.

^c^ Reference: German language region.

^d^ Reference: primary education.

^e^ Reference: no radiotherapy.

^f^ Reference: no HSCT.

^g^ Due to the lower *n* in this analysis, we combined primary and secondary education into in category for children; for adolescents and adults, we kept it separate.

Similar to sun protection, more recent birth cohorts were more likely to report sunburn. Independent of birth cohort, among children, higher age was associated with higher sunburn odds (OR, 1.28; 95% CI, 1.18–1.38). Survivors with migration background were less likely to report sunburn across children and adults. Sunburn was less likely among pediatric (OR, 0.67; 95% CI, 0.43–1.01), adolescent (OR, 0.63; 95% CI, 0.43–0.93), and adult (OR, 0.77; 95% CI, 0.63–0.94) survivors treated with radiotherapy compared to those without, and among adult HSCT recipients (OR, 0.62; 95% CI, 0.39–0.95) compared to non‐HSCT recipients.

### Factors associated with PSE

Pediatric (OR, 3.75; 95% CI, 1.39–9.83), adolescent (OR, 2.06; 95% CI, 0.87–4.55), and adult (OR, 2.07; 95% CI, 1.29–3.27) HSCT recipients were more likely to have had a PSE within the last 12 months compared to non‐HSCT recipients. For radiotherapy, this only applied to pediatric survivors (OR, 2.62; 95% CI, 0.94–6.86) (Table 2). Pediatric (OR, 2.21; 95% CI, 0.99–4.95) and adult (OR, 1.39; 95% CI, 1.06–1.82) survivors in the French or Italian language region were more likely to have had a PSE compared to those in the German language region, as were pediatric survivors whose parents had tertiary education compared to those with primary education. PSE was unrelated to birth year.

### Sensitivity analyses

Across all outcomes, sensitivity analyses yielded similar results. When including skin type, the direction of associations remained unchanged: darker skin type was linked to lower likelihood of sun protection and sunburn but was unrelated to PSE. Results were also stable when replacing birth year with year of study participation, adding season of study participation, or among adult CCS, survivor’s own education (Tables S7–S11).

## DISCUSSION

Sun protection in our study was high: four of five childhood cancer survivors reported to protect always or mostly from sun. Yet, despite good sun protection, one in three experienced a sunburn during the past year. Sun protection was poorer and sunburn more frequent among younger generations. Survivors who received radiotherapy or HSCT did not protect substantially better from the sun than those without these treatments. Only 18% of survivors treated with radiotherapy and 29% of those treated with HSCT reported a physician skin examination (PSE) within the past year, as recommended in the COG guidelines.

Compared with previous CCS studies, sun protection was relatively high in our study. In the North American Childhood Cancer Survivor Study, 67% of predominantly adult survivors reported regular sunscreen use and 41% wore protective clothing,^15^ compared to 77% of adult survivors in our study. Smaller survivor studies reported sun protection prevalences ranging from 23% to 59%.^17^, ^22^, ^38^ Differences may partly be due to measurement, as we assessed sun protection as a composite behavior rather than single practices.^15^, ^17^, ^38^ In the general Swiss population, 54%–80% of children^39^, ^40^, ^41^ and 77% of young adults use sunscreen,^42^ which is slightly lower or similar to our study. Pediatric survivors reported fewer sunburns than children in the general population (40%–60%),^39^, ^40^ likely reflecting greater parental protection after cancer. Sunburn prevalence among adult survivors was comparable to adults in Germany (49%),^43^ possibly due to reduced cancer‐related awareness over time.^44^ Adolescents showed the lowest sun protection and highest sunburn prevalence. With increasing age (i.e., when transitioning into early adolescence) pediatric CCS were less likely to protect from sun and more likely to report sunburn. This pattern is consistent with what we know from the general population, where adolescence represents a vulnerable period for poor health behaviors,^45^ and underscores adolescence as a critical transition period for sun‐related risk behaviors among CCS.

Within each age group, more recently born survivors were less likely to use sun protection and more likely to report sunburn, even if they reported good sun protection. This generational pattern may reflect reduced public awareness and education of skin cancer compared to the 1990s when there was considerable media attention on UV‐related risks,^46^ increased exposure to sunscreen misinformation^47^, ^48^ and tanning‐related appearance norms, amplified by social media.^49^ Female adolescent and adult survivors were more likely to use sun protection, consistent with previous CCS studies^15^, ^18^, ^22^ and broader evidence of greater health consciousness among women.^50^ Survivors with migration background were less likely to use sun protection and to report sunburn if they reported good sun protection. This may be explained by darker skin phototypes and culturally different sun protection education and habits.^20^ Radiotherapy was not associated with better sun protection, in line with earlier findings,^17^ yet was linked to lower sunburn odds in adolescents and adults, as was HSCT among adults. Survivors treated with radiotherapy also reported less frequently sunburn despite good sun protection than those who were not treated with radiotherapy; this points to sun avoidance rather than sun protection.

Only 16% of survivors reported a PSE within the past 12 months, which is slightly higher than the 13% observed in the general Swiss population.^51^ Compliance with the COG’s PSE recommendations was higher among HSCT recipients (29%) than among survivors treated with radiotherapy (17%), the latter aligning with prevalances reported in North American cohorts (11%–31%).^52^, ^53^, ^54^ Low PSE attendance may reflect limited knowledge of prior treatments and associated risks,^52^, ^55^ but also deficiencies in the health care system, including a lack of coordinated and interdisciplinary long‐term follow‐up care programs for adult CCS and uncovered costs for some screening examinations.^56^ To facilitate screening among survivors, low‐threshold approaches, such as easily accessible and cheap or no‐cost community‐based screening in pharmacies, and cancer screening programs accessible to everyone,^57^ may complement structured long‐term follow‐up care programs and enhance screening uptake.^58^

Although still low, higher attendance among HSCT survivors likely reflects their more intensive, multidisciplinary follow‐up care due to greater morbidity^59^, ^60^ and frequent dermatological problems.^61^ Further investigations, such as in interviewing survivors about barriers to PSE, can provide valuable insights. Older age and residence in French‐ or Italian‐speaking regions were associated with higher PSE attendance, possibly reflecting greater health awareness^62^ and regional differences in preventive care cultures.^63^

This study provides comprehensive, population‐based estimates of sun protection, sunburn, and PSE among CCS across the full age spectrum and over two decades of data collection. It extends prior work by assessing adherence to COG recommendations after both radiotherapy and HSCT. Limitations include self‐reporting, which may introduce recall and social desirability bias. Parents who proxy‐reported for children in early adolescent age may not have been fully informed about their child’s sun protection behavior or previous sunburns, which may have resulted in misclassifications. Yet, previous results indicate good agreement between parents and early adolescents regarding sun protection behavior and sunburn.^27^

Incomplete availability of some outcomes and exposures, particularly PSE in children and skin type, limits the study’s statistical power. We did not assess actual sun exposure and skin self‐examination, which the COG recommends monthly and that may reduce melanoma mortality by 63%.^64^ We only inquired about general sun protection, not specifying season of the year. This may have resulted in different reference points regarding the season depending on the time of filling‐in the survey. However, results remained stable after controlling for season of filling‐in the questionnaire. Our cohort is predominantly of Western European ancestry, which may limit generalizability to more diverse populations. Because of a substantial time gap between cancer treatment and study participation, particularly among survivors treated in earlier decades, survivors may not have remembered skin cancer‐related recommendations they received.

Our findings highlight the need for system‐level strategies to embed sun protection and skin cancer screening into survivorship care. Integrating dermatology referrals and annual reminders for radiation or HSCT recipients within survivorship care plans^65^ (e.g., Survivorship Passport) as well as patient or provider activation regarding skin cancer risk after childhood cancer^66^ can improve knowledge and recommendation adherence. Education efforts should emphasize the importance of sun protection for all survivors.^66^

Health care practitioners engaged in follow‐up care should support survivors to attend yearly PSE, particularly after radiotherapy or HSCT, and encourage consistent sun protection, especially among adolescents and male survivors, and younger generations.

## AUTHOR CONTRIBUTIONS

**Carina Nigg**: Conceptualization; methodology; formal analysis; writing—original draft; writing—review and editing; visualization; project administration; funding acquisition. **Maša Žarković:** Writing—review and editing. **Philippa Jörger:** Writing—review and editing. **Eva Maria E. Tinner**: Writing—review and editing. **Calogero Mazzara**: Writing—review and editing. **Eva Brack**: Writing—review and editing. **Paul Castle**: Writing—review and editing. **Alexander Navarini**: Writing—review and editing. **Christina Schindera**: Writing—review and editing. **Claudia E. Kuehni**: Conceptualization; supervision; investigation; writing—review and editing; funding acquisition.

## CONFLICT OF INTEREST STATEMENT

Christina Schindera reports a relationship to Swedish Orphan Biovitrum AB that includes travel reimbursement. This relationship has no association with the current study. The other authors declare no conflicts of interest.

## Supporting information

Supplementary Material

## Data Availability

The data that support the findings of this study are available on request from the corresponding author. The data are not publicly available due to privacy or ethical restrictions.
