# Supplementary material for: Sun protection and skin cancer screening after childhood cancer—A report from the Swiss Childhood Cancer Survivor Study
Source: Cancer. 2026 Mar 29;132(7):e70364. doi: 10.1002/cncr.70364 (PMC13033617; doi:10.1002/cncr.70364)
Supplement: Supplementary file 1 — Supplementary Material [file CNCR-132-e70364-s001.docx]

**Supplementary material**

**Sun protection and skin cancer screening after childhood cancer – a report from the Swiss Childhood Cancer Survivor Study (SCCSS)**

Carina Nigg, PhD, Maša Žarković, MD, Philippa Jörger, MSc, Eva Maria E. Tinner, MD, Calogero Mazzara, MD, Eva Brack, MD, PhD, Paul Castle, Alexander Navarini, MD, Christina Schindera, MD, PhD, Claudia E Kuehni, MD

**Corresponding author:**

Prof. Claudia E. Kuehni, MD, MSc

Institute of Social and Preventive Medicine

University of Bern

Mittelstrasse 43

3012 Bern

Switzerland

E-mail: claudia.kuehni@unibe.ch

**Table of content**

[**Supplementary Figure 1.** Study population tree of the SCCSS study on sun protection behaviors and physician skin examination 3](#_Toc220131721)

[**Supplementary Table 1.** Overview sun protection behaviors and physician skin examination questions, response options, and categorization 4](#_Toc220131722)

[**Supplementary Table 2.** Model fit indices of multivariable logistic regression models including birth year compared to multivariable logistic regression models including year of study 5](#_Toc220131723)

[**Supplementary Table 3.** Prevalence of sun protection, sunburn, and physician skin examinations among childhood cancer survivors 6](#_Toc220131724)

[**Supplementary Table 4.** Comparison of childhood cancer survivors who report good sun protection and did not have a sunburn and those who report good sun protection still had a sunburn 7](#_Toc220131725)

[**Supplementary Table 5.** Proportion of childhood cancer survivors who attended a physician skin examination within the last 12 months as recommended by the Children’s Oncology Group 2023 Follow-Up Care Guidelines stratified by radiotherapy and HSCT exposure 8](#_Toc220131726)

[**Supplementary Table 6.** Factors associated with sun protection, sunburn, and physician skin examination in modified Poisson regression in child, adolescent, and adult childhood cancer survivors 9](#_Toc220131727)

[**Supplementary Table 7.** Skin type descriptives of pediatric, adolescent, and adult childhood cancer survivors 10](#_Toc220131728)

[**Supplementary Table 8.** Sensitivity analysis: Factors associated with sun protection, sunburn, and physician skin examinations in multivariable logistic regression in child, adolescent, and adult childhood cancer survivors, including skin type 11](#_Toc220131729)

[**Supplementary Table 9.** Sensitivity analysis: Factors associated with sun protection, sunburn, and physician skin examinations in multivariable logistic regression in child, adolescent, and adult childhood cancer survivors, including year of study instead of birth year as exposure 12](#_Toc220131730)

[**Supplementary Table 10.** Sensitivity analysis: Factors associated with sun protection and sunburn in multivariable logistic regression in child, adolescents, and adult childhood cancer survivors, including season 13](#_Toc220131731)

[**Supplementary Table 11.** Sensitivity analysis: Factors associated with sun protection, sunburn and physician skin examination in multivariable logistic regression in adult childhood cancer survivors, including their own highest education obtained at the time of study 14](#_Toc220131732)

# **Supplementary Figure 1.** Study population tree of the SCCSS study on sun protection behaviors and physician skin examination


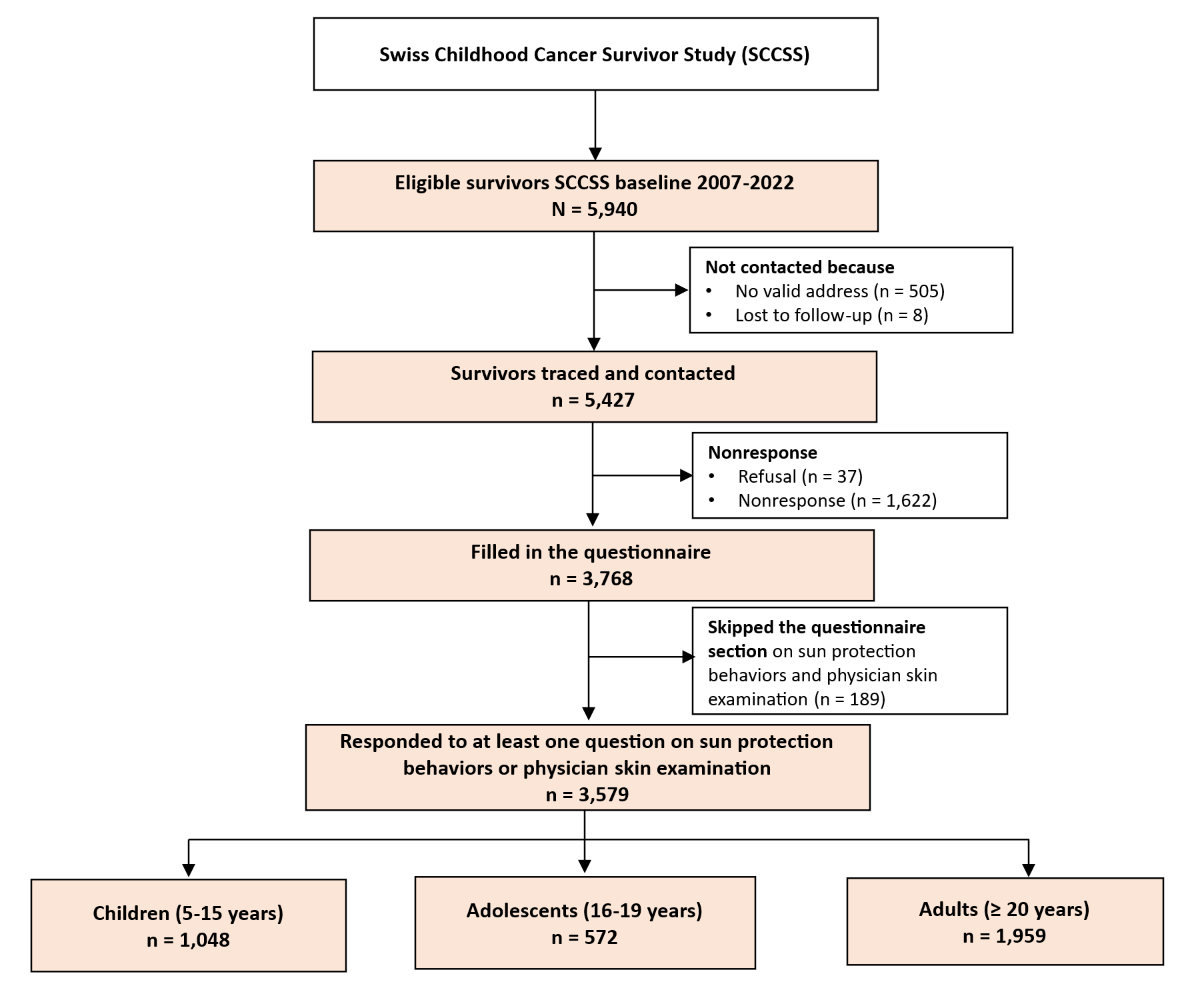


# **Supplementary Table 1.** Overview sun protection behaviors and physician skin examination questions, response options, and categorization

| **Outcome variables** | **Question** | **Response option** | **Coding for regression analysis** |
| --- | --- | --- | --- |
| Sun protection | How consequently to you protect yourself (for parent questionnaire: your child) from sun? For example, applying sunscreen, wearing clothing and/or a hat, shading, considering day time for one’s sun exposure | 1: Always  2: Most of the time  3: Every now and then  4: Never | 1 and 2: Yes  3 and 4: No |
| Sunburn | Have you (for parent questionnaire: your child) had one or more sunburns the previous summer? | 1: No  2: Yes | 1: No  2: Yes |
| Physician skin examinations | Have you (your child) ever had your skin or moles medically examined? | 1: Yes, less than 12 months ago  2: Yes, more than 12 months ago  3: No, never | 1 and 2: Yes  3: No |

*Please note: Not all questions were included in all surveys or in all age groups. ^a^ In earlier surveys, response options were only “yes” and “no”*

# **Supplementary Table 2.** Model fit indices of multivariable logistic regression models including birth year compared to multivariable logistic regression models including year of study

| **Model** | **AIC** | **BIC** |
| --- | --- | --- |
| **Children (5-15 years)** |  |  |
| Sun protection with birth year | 658 | 708 |
| Sun protection with year of study | 659 | 713 |
| Sunburn with birth year | 1,055 | 1,104 |
| Sunburn with year of study | 1,057 | 1,111 |
| PSE with birth year | 200 | 232 |
| PSE with study year | 201 | 230 |
| **Adolescents (16-19 years)** |  |  |
| Sun protection with birth year | 593 | 635 |
| Sun protection with year of study | 584 | 630 |
| Sunburn with birth year | 753 | 796 |
| Sunburn with year of study | 756 | 808 |
| PSE with birth year | 466 | 509 |
| PSE with year of study | 470 | 521 |
| **Adults (≥ 20 years)** |  |  |
| Sun protection with birth year | 1,675 | 1,729 |
| Sun protection with year of study | 1,676 | 1,736 |
| Sunburn with birth year | 2,371 | 2,426 |
| Sunburn with year of study | 2,375 | 2,441 |
| PSE with birth year | 1,577 | 1,632 |
| PSE with year of study | 1,579 | 1,645 |

# **Supplementary Table 3.** Prevalence of sun protection, sunburn, and physician skin examinations among childhood cancer survivors

|  | **Children (5-15 years)** | | **Adolescents (16-19 years)** | | **Adults (≥ 20 years)** | | **Overall** | |
| --- | --- | --- | --- | --- | --- | --- | --- | --- |
|  | **N** | **95%CI** | **N** | **95%CI** | **N** | **95%CI** | **N** | **95%CI** |
| *Sun protection* | **n = 1,048** |  | **n = 499** |  | **n = 1,727** |  | **n = 3,274** |  |
| Always | 390 (37%) | 34-40 | 78 (16%) | 13-19 | 464 (27%) | 25-29 | 932 (28%) | 27-30 |
| Mostly | 535 (51%) | 48-54 | 245 (49%) | 45-53 | 862 (50%) | 48-52 | 1642 (50%) | 48-52 |
| Sometimes | 91 (9%) | 7-11 | 111 (22%) | 19-26 | 285 (17%) | 15-18 | 487 (15%) | 14-16 |
| (Almost) never | 26 (3%) | 2-4 | 63 (13%) | 10-16 | 105 (6%) | 5-7 | 194 (6%) | 5-7 |
| *Missing* | *6 (1%)* |  | *2 (<1%)* |  | *11 (1%)* |  | *19 (<1%)* |  |
|  |  |  |  |  |  |  |  |  |
| *Sun protection (dichotomized)* | **n = 1,048** |  | **n = 499** |  | **n = 1,727** |  | **n = 3,274** |  |
| No | 117 (11%) | 9-13 | 174 (35%) | 31-39 | 390 (23%) | 21-25 | 681 (21%) | 19-22 |
| Yes | 925 (89%) | 86-90 | 323 (65%) | 60-69 | 1326 (77%) | 75-79 | 2574 (79%) | 77-80 |
| *Missing* | *6 (1%)* |  | *2 (0.4%)* |  | *11 (1%)* |  | *19 (<1%)* |  |
|  |  |  |  |  |  |  |  |  |
| *Sunburn(s) last summer* | **n = 1,048** |  | **n= 572** |  | **n = 1,959** |  | **n = 3,579** |  |
| No | 800 (77%) | 74-79 | 291 (51%) | 47-55 | 1109 (57%) | 54-59 | 2200 (62%) | 60-63 |
| Yes | 241 (23%) | 21-26 | 275 (49%) | 44-52 | 829 (43%) | 40-45 | 1345 (38%) | 36-39 |
| *Missing* | *7 (1%)* |  | *6 (1 %)* |  | *21 (1%)* |  | *34 (1%)* |  |
|  |  |  |  |  |  |  |  |  |
| *Physician skin examination* | **n= 284** |  | **n= 572** |  | **n = 1,959** |  | **n = 2,815** |  |
| Yes, < 12 months ago | 36 (13%) | 9-17 | 87 (16%) | 12-18 | 315 (16%) | 15-18 | 438 (16%) | 14-17 |
| Yes, ≥ 12 months ago | 44 (16%) | 12-20 | 142 (25%) | 21-29 | 642 (33%) | 31-25 | 828 (30%) | 28-31 |
| No, never | 202 (72%) | 66-76 | 331 (59%) | 54-62 | 952 (50%) | 46-51 | 1485 (54%) | 51-55 |
| *Missing* | *2 (1%)* |  | *12 (2%)* |  | *50 (3%)* |  | *64 (2%)* |  |
|  |  |  |  |  |  |  |  |  |
| *Physician skin examination within the last 12 months* | **n= 284** |  | **n= 572** |  | **n = 1,959** |  | **n = 2,815** |  |
| No | 246 (87%) | 82-90 | 473 (84%) | 79-86 | 1594 (84%) | 80-83 | 2313 (82%) | 81-84 |
| Yes | 36 (13%) | 9-17 | 87 (16%) | 12-18 | 315 (16%) | 15-18 | 438 (16%) | 14-17 |
| *Missing* | *2 (1%)* |  | *12 (2%)* |  | *50 (3%)* |  | *64 (2%)* |  |

Please note: Not all questions were asked in all surveys or in all age categories so that we report the available *n* for each variable.

# **Supplementary Table 4.** Comparison of childhood cancer survivors who report good sun protection and did not have a sunburn and those who report good sun protection still had a sunburn

|  | **Children (5-15 years)** | | |  | **Adolescents (16-19 years)** | | |  | **Adults (≥20 years)** | | |
| --- | --- | --- | --- | --- | --- | --- | --- | --- | --- | --- | --- |
|  | **Protect &**  **no sunburn**  **N = 685** | **Protect &**  **sunburn**  **N = 214** | *p* |  | **Protect &**  **no sunburn**  **N = 164** | **Protect &**  **sunburn**  **N = 143** | *p* |  | **Protect &**  **no sunburn**  **N = 733** | **Protect &**  **sunburn**  **N = 517** | *p* |
| **Socio-demographic characteristics** |  |  |  |  |  |  |  |  |  |  |  |
| *Age at time of questionnaire* |  |  | *0.003* |  |  |  | *0.423* |  |  |  | *<0.001* |
| Median [IQR] | 12 [10-14] | 12 [11-14] |  |  | 18 [17-19] | 18 [17-19] |  |  | 29 [24-35] | 26 [23-31] |  |
| Mean (SD) | 12 (2.7) | 12 (2.4) |  |  | 18 (1.2) | 18 (1.2) |  |  | 30 (7.6) | 28 (6.4) |  |
| *Year of birth* |  |  | *0.002* |  |  |  | *0.195* |  |  |  | *<0.001* |
| Median [IQR] | 2003  [1998-2007] | 2005  [2000-2008] |  |  | 1992  [1990-1993] | 1992  [1990-1993] |  |  | 1980  [1974-1985] | 1984  [178-1988] |  |
| Mean (SD) | 2003(5.1) | 2004 (4.9) |  |  | 1992 (3.5) | 1993 (4.3) |  |  | 1979 (7.9) | 1984 (8.0) |  |
| *Year of study participation,*  *N (%)* |  |  | *<0.001* |  |  |  | *0.239* |  |  |  | *<0.001* |
| 2007-2013 | 321 (82.7%) | 67 (17.3%) |  |  |  |  |  |  |  |  |  |
| 2007-2009 |  |  |  |  | 99 (55%) | 81 (45%) |  |  | 494 (63%) | 296 (38%) |  |
| 2010-2013 |  |  |  |  | 51 (55%) | 41 (45%) |  |  | 184 (60%) | 121 (40%) |  |
| 2015-2017 | 218 (78.4%) | 60 (21.6%) |  |  |  |  |  |  |  |  |  |
| 2021-2022 | 146 (62.7%) | 87 (37.3%) |  |  | 14 (40%) | 21 (60%) |  |  | 55 (36%) | 100 (65%) |  |
| *Sex, N (%)* |  |  | *1.0* |  |  |  | *0.337* |  |  |  | *1.0* |
| Female | 311 (76%) | 97 (24%) |  |  | 84 (51%) | 82 (50%) |  |  | 375 (59%) | 265 (41%) |  |
| Male | 374 (76%) | 117 (24%) |  |  | 80 (57%) | 61 (43%) |  |  | 358 (59%) | 252 (41%) |  |
| *Migration background,*  *N (%)* |  |  | *0.060* |  |  |  | *0.387* |  |  |  | *<0.001* |
| No | 544 (75%) | 183 (25%) |  |  | 134 (52%) | 123 (48%) |  |  | 597 (56%) | 469 (44%) |  |
| Yes | 141 (82%) | 31 (18%) |  |  | 30 (60%) | 20 (40%) |  |  | 136 (74%) | 48 (26%) |  |
| *Language, N (%)* |  |  | *0.784* |  |  |  | *0.736* |  |  |  | *0.221* |
| German | 468 (76%) | 149 (24%) |  |  | 122 (54%) | 103 (46%) |  |  | 507 (58%) | 375 (43%) |  |
| French/Italian | 217 (77%) | 65 (23%) |  |  | 42 (51%) | 40 (49%) |  |  | 226 (61%) | 142 (39%) |  |
| *Parental highest education*  *N (%)* |  |  | *0.641* |  |  |  | *0.716* |  |  |  | *<0.001* |
| Primary | 49 (80%) | 12 (20%) |  |  | 9 (56%) | 7 (44%) |  |  | 93 (76%) | 29 (24%) |  |
| Secondary | 242 (75%) | 81 (25%) |  |  | 107 (55%) | 88 (45%) |  |  | 403 (59%) | 284 (41%) |  |
| Tertiary | 394 (77%) | 121 (23%) |  |  | 48 (50%) | 48 (50%) |  |  | 237 (54%) | 204 (46%) |  |
| **Cancer-related characteristics** |  |  |  |  |  |  |  |  |  |  |  |
| *Age at diagnosis* |  |  | *0.047* |  |  |  | *0.110* |  |  |  | *0.738* |
| Median [IQR] | 3 [1-5] | 3 [2-5] |  |  | 6 [3-10] | 5 [2-9] |  |  | 12 [6-15] | 12 [5-15] |  |
| Mean (SD) | 3 (2.4) | 4 (2.4) |  |  | 7 (3.8) | 6 (3.9) |  |  | 11 (5.6) | 11 (5.9) |  |
| *Time since diagnosis* |  |  | *0.234* |  |  |  | *0.056* |  |  |  | *<0.001* |
| Median [IQR] | 8 [7-10] | 9 [7-10] |  |  | 11 [8-14] | 12 [9-15] |  |  | 20 [14-25] | 17 [11-22] |  |
| Mean (SD) | 9 (2.1) | 9 (2.1) |  |  | 11 (3.7) | 12 (3.8) |  |  | 20 (7.7) | 17 (7.2) |  |
| *Year of diagnosis* |  |  | *<0.001* |  |  |  | *0.913* |  |  |  | *<0.001* |
| Median [IQR] | 2006  [2002-2010] | 2009  [2004-2012] |  |  | 1998  [1995-2002] | 1997  [1994-2002] |  |  | 1989  [1984-1996] | 1993  [1986-2001] |  |
| Mean (SD) | 2006 (5.0) | 2008 (5.3) |  |  | 1999 (5.6) | 1999 (6.7) |  |  | 1990 (9.0) | 1994 (10.2) |  |
| *Radiotherapy, N (%)* |  |  | *0.028* |  |  |  | *0.020* |  |  |  | *<0.001* |
| No | 544 (75%) | 185 (25%) |  |  | 100 (49%) | 106 (52%) |  |  | 395 (53%) | 346 (47%) |  |
| Yes | 141 (83%) | 29 (17%) |  |  | 64 (63%) | 37 (37%) |  |  | 338 (66%) | 171 (34%) |  |
| *Chemotherapy, N (%)* |  |  | *0.586* |  |  |  | *0.697* |  |  |  | *1.0* |
| No | 118 (74%) | 41 (26%) |  |  | 26 (50%) | 26 (50%) |  |  | 178 (59%) | 125 (41%) |  |
| Yes | 567 (77%) | 173 (23%) |  |  | 138 (54%) | 117 (46%) |  |  | 555 (59%) | 392 (41%) |  |
| *HSCT, N (%)* |  |  | *0.181* |  |  |  | *0.218* |  |  |  | *0.154* |
| No | 625 (76%) | 202 (24%) |  |  | 156 (55%) | 130 (45%) |  |  | 687 (58%) | 495 (42%) |  |
| Yes | 60 (83%) | 12 (17%) |  |  | 8 (38%) | 13 (62%) |  |  | 46 (68%) | 22 (32%) |  |

*Please note: percentages present row percentages*

# **Supplementary Table 5.** Proportion of childhood cancer survivors who attended a physician skin examination within the last 12 months as recommended by the Children’s Oncology Group 2023 Follow-Up Care Guidelines stratified by radiotherapy and HSCT exposure

|  | **Total N^a^** | **N^b^** | **%^c^** | **95% CI** | **p^d^** |
| --- | --- | --- | --- | --- | --- |
|  | **Children** | | | |  |
| Radiotherapy | 43 | 9 | 21 | 8-34 | **0.023** |
| HSCT | 28 | 10 | 36 | 17-55 | **<0.001** |
| No radiotherapy or HSCT | 221 | 20 | 9 | 5-13 |  |
|  | **Adolescents** | | | |  |
| Radiotherapy | 160 | 28 | 18 | 12-23 | 0.280 |
| HSCT | 36 | 10 | 28 | 12-43 | **0.026** |
| No radiotherapy or HSCT | 382 | 53 | 14 | 10-17 |  |
|  | **Adults** | | | |  |
| Radiotherapy | 723 | 125 | 17 | 15-20 | 0.376 |
| HSCT | 111 | 31 | 28 | 20-36 | **0.001** |
| No radiotherapy or HSCT | 1144 | 180 | 16 | 14-18 |  |
|  | **Overall** | | | |  |
| Radiotherapy | 926 | 162 | 17 | 15-20 | **0.041** |
| HSCT | 175 | 51 | 29 | 22-36 | **<0.001** |
| No radiotherapy or HSCT | 1747 | 253 | 14 | 13-16 |  |

^a^ Total N corresponding to the number of CCS responding to the question on skin examinations with(out) this treatment

^b^ Number of CCS reporting to have attended a preventive skin examination within the last 12 months

^c^ % of CCS who attended a preventive skin exam within the last 12 months

^d^ p-level of chi-square test comparing those received treatment and those who did not receive treatment

HSCT = hematopoietic stem cell transplantation

# **Supplementary Table 6.** Factors associated with sun protection, sunburn, and physician skin examination in modified Poisson regression in child, adolescent, and adult childhood cancer survivors

|  | **Children (5-15 years)** | | | | **Adolescents (16-19 years)** | | | | **Adults (≥20 years)** | | |
| --- | --- | --- | --- | --- | --- | --- | --- | --- | --- | --- | --- |
|  | *RR* | *95%CI* | *p* |  | *RR* | *95%CI* | *p* |  | *RR* | *95%CI* | *p* |
|  | **Sun protection** | | | | | | | | | | |
|  | **N = 1,013** | | | | **N = 476** | | | | **N = 1,626** | | |
| Age at study | 0.97 | 0.96;0.98 | <0.001 |  | 0.97 | 0.92;1.03 | 0.301 |  | 1.00 | 0.99;1.01 | 0.967 |
| Male^a^ | 0.97 | 0.93;1.01 | 0.143 |  | 0.73 | 0.64;0.84 | <0.001 |  | 0.87 | 0.82;0.91 | <0.001 |
| Migration background^b^ | 0.95 | 0.89;1.01 | 0.080 |  | 0.83 | 0.66;1.03 | 0.093 |  | 0.87 | 0.79;0.95 | 0.001 |
| French/Italian language region^c^ | 1.02 | 0.98;1.07 | 0.350 |  | 1.02 | 0.88;1.18 | 0.783 |  | 1.00 | 0.94;1.06 | 0.967 |
| Parental education^d^ |  |  |  |  |  |  |  |  |  |  |  |
| Secondary | 1.12 | 1.00;1.26 | 0.059 |  | 1.13 | 0.78;1.64 | 0.522 |  | 1.06 | 0.96;1.18 | 0.223 |
| Tertiary | 1.12 | 1.00;1.26 | 0.058 |  | 1.27 | 0.86;1.85 | 0.226 |  | 1.10 | 0.99;1.22 | 0.065 |
| Radiotherapy^e^ | 1.03 | 0.98;1.09 | 0.287 |  | 1.15 | 1.01;1.31 | 0.037 |  | 1.04 | 0.99;1.10 | 0.146 |
| HSCT^f^ | 1.01 | 0.93;1.08 | 0.874 |  | 1.14 | 0.92;1.42 | 0.226 |  | 1.00 | 0.90;1.12 | 0.961 |
| Birth year | 0.99 | 0.99;1.00 | 0.048 |  | 0.98 | 0.96;1.00 | 0.068 |  | 0.99 | 0.98;1.00 | 0.007 |
|  | **Sunburn** | | | | | | | | | | |
|  | **N = 1,013** | | | | **N = 542** | | | | **N = 1,837** | | |
| Age at study | 1.20 | 1.13;1.26 | <0.001 |  | 1.01 | 0.94;1.09 | 0.730 |  | 1.00 | 0.99;1.01 | 0.971 |
| Male^a^ | 0.99 | 0.80;1.22 | 0.914 |  | 0.85 | 0.72;1.02 | 0.075 |  | 1.02 | 0.93;1.13 | 0.636 |
| Migration background^b^ | 0.63 | 0.46;0.87 | 0.005 |  | 0.84 | 0.65;1.10 | 0.212 |  | 0.65 | 0.54;0.78 | <0.001 |
| French/Italian language region^c^ | 0.96 | 0.76;1.22 | 0.754 |  | 1.05 | 0.87;1.27 | 0.603 |  | 0.92 | 0.82;1.03 | 0.164 |
| Parental education^d^ |  |  |  |  |  |  |  |  |  |  |  |
| Secondary | 1.06 | 0.66;1.71 | 0.802 |  | 1.05 | 0.66;1.68 | 0.837 |  | 1.12 | 0.89;1.41 | 0.343 |
| Tertiary | 0.93 | 0.58;1.49 | 0.766 |  | 1.16 | 0.72;1.88 | 0.532 |  | 1.25 | 0.99;1.59 | 0.058 |
| Radiotherapy^e^ | 0.74 | 0.54;1.02 | 0.068 |  | 0.79 | 0.63;0.98 | 0.029 |  | 0.86 | 0.77;0.96 | 0.010 |
| HSCT^f^ | 0.69 | 0.41;1.17 | 0.165 |  | 1.19 | 0.88;1.60 | 0.259 |  | 0.76 | 0.58;1.00 | 0.047 |
| Birth year | 1.10 | 1.07;1.14 | <0.001 |  | 1.02 | 1.00;1.04 | 0.088 |  | 1.03 | 1.02;1.04 | <0.001 |
|  | **Physician skin examination** | | | | | | | | | | |
|  | **N = 275** | | | | **N = 538** | | | | **N = 1,807** | | |
| Age at study | 0.34 | 0.11;1.06 | 0.063 |  | 1.01 | 0.87;1.19 | 0.861 |  | 1.03 | 1.01;1.06 | 0.018 |
| Male^a^ | 0.96 | 0.54;1.73 | 0.899 |  | 1.32 | 0.87;1.99 | 0.190 |  | 0.70 | 0.57;0.86 | 0.001 |
| Migration background^b^ | 0.92 | 0.41;2.06 | 0.835 |  | 1.23 | 0.74;2.04 | 0.421 |  | 1.27 | 0.97;1.67 | 0.081 |
| French/Italian language region^c^ | 1.85 | 1.01;3.42 | 0.048 |  | 1.07 | 0.69;1.64 | 0.762 |  | 1.31 | 1.05;1.63 | 0.017 |
| Parental education^g^ |  |  |  |  |  |  |  |  |  |  |  |
| Secondary |  |  |  |  | 1.45 | 0.54;3.85 | 0.461 |  | 0.96 | 0.68;1.37 | 0.839 |
| Tertiary | 2.70 | 1.03;7.11 | 0.044 |  | 1.47 | 0.54;4.04 | 0.451 |  | 1.22 | 0.85;1.74 | 0.281 |
| Radiotherapy^e^ | 2.16 | 1.10;4.26 | 0.026 |  | 1.13 | 0.72;1.76 | 0.601 |  | 1.01 | 0.81;1.25 | 0.935 |
| HSCT^f^ | 2.61 | 1.39;4.89 | 0.003 |  | 1.79 | 0.95;3.34 | 0.070 |  | 1.75 | 1.24;2.46 | 0.002 |
| Birth year | 0.39 | 0.12;1.21 | 0.103 |  | 1.00 | 0.95;1.06 | 0.923 |  | 1.01 | 0.99;1.03 | 0.331 |

^a^ reference: female, ^b^ reference: no migration background, ^c^ reference: German language region, ^d^ reference: primary education, ^e^ reference: no radiotherapy; ^f^ reference: no HSCT, ^g^ due to the lower n in this analysis, we combined primary and secondary education into in category for children; for adolescents and adults, we kept it separate, HSCT = hematopoietic stem cell transplantation

# **Supplementary Table 7.** Skin type descriptives of pediatric, adolescent, and adult childhood cancer survivors

|  | **Children (5-15 years)**  **n = 598** | **Adolescents (16-19 years)**  **n = 572** | **Adults (≥ 20 years)**  **n = 470** | **Overall**  **N = 1,640** |
| --- | --- | --- | --- | --- |
| *Skin type 5 levels* |  |  |  |  |
| Skin type 1 | 25 (4%) | 18 (3%) | 20 (4%) | 63 (4%) |
| Skin type 2 | 159 (27%) | 142 (25%) | 125 (27%) | 426 (26%) |
| Skin type 3 | 282 (48%) | 285 (51%) | 237 (51%) | 804 (49%) |
| Skin type 4 | 112 (19%) | 107 (19%) | 80 (17%) | 299 (18%) |
| Skin type 5 | 14 (2%) | 12 (2%) | 5 (1%) | 31 (2%) |
| *Missing* | *6 (1%)* | *8 (1%)* | *3 (1%)* | *17 (1%)* |
|  |  |  |  |  |
| *Skin type 3 levels (for analysis)* |  |  |  |  |
| Skin type 1-2 | 184 (31%) | 160 (28%) | 145 (31%) | 489 (30%) |
| Skin type 3 | 282 (48%) | 285 (51%) | 237 (51%) | 804 (50%) |
| Skin type 4-5 | 126 (21%) | 119 (21%) | 85 (18%) | 330 (20%) |
| *Missing* | *6 (1%)* | *8 (1%)* | *3 (1%)* | *17 (1%)* |

# **Supplementary Table 8.** Sensitivity analysis: Factors associated with sun protection, sunburn, and physician skin examinations in multivariable logistic regression in child, adolescent, and adult childhood cancer survivors, including skin type

|  | **Children (5-15 years)** | | | | **Adolescents (16-19 years)** | | | | **Adults (≥20 years)** | | | |
| --- | --- | --- | --- | --- | --- | --- | --- | --- | --- | --- | --- | --- |
|  | *OR* | *95%CI* | *p* |  | *OR* | *95%CI* | *p* |  | *OR* | *95%CI* | *p* | |
|  | **Sun protection** | | | | | | | | | | | |
|  | **N = 576** | | | | **N = 470** | | | | **N = 224** | | | |
| Age at study | 0.80 | 0.71;0.89 | **<0.001** |  | 0.95 | 0.80;1.13 | 0.538 |  | 1.03 | 0.98;1.09 | 0.224 | |
| Male^a^ | 0.97 | 0.56;1.67 | 0.908 |  | 0.42 | 0.27;0.64 | **<0.001** |  | 0.66 | 0.34;1.25 | 0.203 | |
| Migration background^b^ | 0.80 | 0.42;1.55 | 0.492 |  | 0.66 | 0.38;1.15 | 0.142 |  | 0.37 | 0.15;0.93 | **0.035** | |
| French/Italian language region^c^ | 0.84 | 0.46;1.58 | 0.573 |  | 0.87 | 0.55;1.40 | 0.572 |  | 1.56 | 0.80;3.16 | 0.202 | |
| Parental education^d^ |  |  |  |  |  |  |  |  |  |  |  | |
| Secondary | 2.71 | 0.98;7.30 | 0.050 |  | 1.01 | 0.43;2.39 | 0.973 |  | 0.93 | 0.26;3.13 | 0.912 | |
| Tertiary | 1.89 | 0.73;4.65 | 0.176 |  | 1.31 | 0.53;3.21 | 0.559 |  | 1.29 | 0.37;4.24 | 0.682 | |
| Radiotherapy^e^ | 2.14 | 0.96;5.47 | 0.083 |  | 1.71 | 1.08;2.74 | **0.025** |  | 1.55 | 0.78;3.18 | 0.215 | |
| HSCT^f^ | 0.84 | 0.32;2.64 | 0.746 |  | 1.53 | 0.64;4.03 | 0.361 |  | 0.71 | 0.21;2.50 | 0.578 | |
| Skin type (ref. 1-2) |  |  |  |  |  |  |  |  |  |  |  | |
| Skin type 3 | 0.25 | 0.10;0.57 | **0.002** |  | 0.48 | 0.28;0.81 | **0.008** |  | 0.59 | 0.28;1.23 | 0.166 | |
| Skin type 4-6 | 0.12 | 0.04;0.28 | **<0.001** |  | 0.24 | 0.12;0.44 | **<0.001** |  | 0.21 | 0.09;0.51 | **0.001** | |
|  | **Sunburn** | | | | | | | | | | | |
|  | **N = 576** | | | | **N = 537** | | | | **N = 445** | | | |
| Age at study | 1.14 | 1.06;1.23 | **0.001** |  | 1.02 | 0.88;1.18 | 0.806 |  | 0.93 | 0.91;0.96 | **<0.001** | |
| Male^a^ | 1.03 | 0.70;1.52 | 0.866 |  | 0.82 | 0.57;1.18 | 0.286 |  | 1.05 | 0.70;1.57 | 0.819 | |
| Migration background^b^ | 0.60 | 0.36;0.99 | 0.050 |  | 0.93 | 0.56;1.55 | 0.783 |  | 0.40 | 0.22;0.72 | **0.002** | |
| French/Italian language region^c^ | 0.71 | 0.46;1.09 | 0.118 |  | 0.87 | 0.58;1.30 | 0.503 |  | 1.19 | 0.77;1.86 | 0.433 | |
| Parental education^d^ |  |  |  |  |  |  |  |  |  |  |  |  |
| Secondary | 1.00 | 0.45;2.35 | 0.992 |  | 0.81 | 0.34;1.93 | 0.628 |  | 0.82 | 0.37;1.82 | 0.622 | |
| Tertiary | 0.67 | 0.30;1.53 | 0.330 |  | 1.02 | 0.42;2.47 | 0.973 |  | 1.18 | 0.53;2.62 | 0.685 | |
| Radiotherapy^e^ | 0.50 | 0.28;0.88 | **0.019** |  | 0.57 | 0.38;0.85 | **0.006** |  | 1.03 | 0.67;1.59 | 0.879 | |
| HSCT^f^ | 0.46 | 0.18;1.02 | 0.073 |  | 1.61 | 0.78;3.42 | 0.203 |  | 0.77 | 0.35;1.67 | 0.503 | |
| Skin type (ref. 1-2) |  |  |  |  |  |  |  |  |  |  |  |  |
| Skin type 3 | 0.57 | 0.37;0.87 | **0.009** |  | 0.60 | 0.39;0.91 | **0.017** |  | 0.99 | 0.62;1.57 | 0.959 | |
| Skin type 4-6 | 0.19 | 0.10;0.36 | **<0.001** |  | 0.16 | 0.09;0.28 | **<0.001** |  | 0.47 | 0.26;0.84 | **0.012** | |
|  | **Physician skin examination** | | | | | | | | | | | |
|  | **N = 270** | | | | **N = 531** | | | | **N = 442** | | | |
| Age at study | 0.85 | 0.72;0.99 | **0.044** |  | 1.01 | 0.83;1.23 | 0.935 |  | 1.02 | 0.98;1.05 | 0.307 | |
| Male^a^ | 1.00 | 0.46;2.18 | 0.995 |  | 1.39 | 0.85;2.30 | 0.190 |  | 0.49 | 0.29;0.83 | **0.009** | |
| Migration background^b^ | 0.94 | 0.36;2.26 | 0.890 |  | 1.44 | 0.74;2.68 | 0.270 |  | 1.07 | 0.49;2.22 | 0.859 | |
| French/Italian language region^c^ | 2.43 | 1.06;5.61 | **0.036** |  | 0.99 | 0.57;1.69 | 0.983 |  | 1.18 | 0.67;2.05 | 0.557 | |
| Parental education^g^ |  |  |  |  |  |  |  |  |  |  |  |  |
| Secondary |  |  |  |  | 1.49 | 0.49;5.64 | 0.510 |  | 0.74 | 0.29;2.06 | 0.545 | |
| Tertiary | 4.10 | 1.47;13.92 | **0.012** |  | 1.47 | 0.47;5.67 | 0.536 |  | 0.89 | 0.35;2.48 | 0.821 | |
| Radiotherapy^e^ | 2.55 | 0.91;6.78 | 0.064 |  | 1.12 | 0.66;1.88 | 0.663 |  | 1.00 | 0.57;1.74 | 0.993 | |
| HSCT^f^ | 3.63 | 1.33;9.56 | **0.010** |  | 2.02 | 0.85;4.44 | 0.091 |  | 1.88 | 0.72;4.50 | 0.170 | |
| Skin type (ref. 1-2) |  |  |  |  |  |  |  |  |  |  |  |  |
| Skin type 3 | 0.57 | 0.23;1.41 | 0.219 |  | 0.59 | 0.34;1.04 | 0.066 |  | 0.95 | 0.54;1.70 | 0.866 | |
| Skin type 4-6 | 1.40 | 0.49;3.98 | 0.526 |  | 0.67 | 0.33;1.33 | 0.260 |  | 0.49 | 0.19;1.11 | 0.101 | |

^a^ reference: female, ^b^ reference: no migration background, ^c^ reference: German language region, ^d^ reference: primary education, ^e^ reference: no radiotherapy; ^f^ reference: no HSCT, ^g^ due to the lower n in this analysis, we combined primary and secondary education into in category for children; for adolescents and adults, we kept it separate, HSCT = hematopoietic stem cell transplantation

# **Supplementary Table 9.** Sensitivity analysis: Factors associated with sun protection, sunburn, and physician skin examinations in multivariable logistic regression in child, adolescent, and adult childhood cancer survivors, including year of study instead of birth year as exposure

|  | **Children (5-15 years)** | | | | **Adolescents (16-19 years)** | | | | **Adults (≥20 years)** | | | |
| --- | --- | --- | --- | --- | --- | --- | --- | --- | --- | --- | --- | --- |
|  | *OR* | *95%CI* | *p* |  | *OR* | *95%CI* | *p* |  | *OR* | *95%CI* | *p* | |
|  | **Sun protection** | | | | | | | | | | | |
|  | **N = 1,013** | | | | **N = 476** | | | | **N = 1,626** | | | |
| Age at study | 0.78 | 0.71;0.85 | **<0.001** |  | 0.95 | 0.80;1.13 | 0.558 |  | 1.04 | 1.02;1.07 | **<0.001** | |
| Male^a^ | 0.72 | 0.47;1.09 | 0.125 |  | 0.37 | 0.24;0.56 | **<0.001** |  | 0.51 | 0.40;0.65 | **<0.001** | |
| Migration background^b^ | 0.63 | 0.38;1.05 | 0.069 |  | 0.58 | 0.34;0.98 | **0.043** |  | 0.58 | 0.43;0.80 | **0.001** | |
| French/Italian language region^c^ | 1.28 | 0.82;2.04 | 0.293 |  | 1.19 | 0.75;1.90 | 0.461 |  | 1.01 | 0.77;1.33 | 0.947 | |
| Parental education^d^ |  |  |  |  |  |  |  |  |  |  |  | |
| Secondary | 1.96 | 0.97;3.89 | 0.057 |  | 1.25 | 0.53;2.95 | 0.602 |  | 1.30 | 0.87;1.93 | 0.197 | |
| Tertiary | 1.95 | 0.97;3.79 | 0.054 |  | 1.71 | 0.69;4.22 | 0.243 |  | 1.55 | 1.01;2.36 | **0.042** | |
| Radiotherapy^e^ | 1.33 | 0.77;2.43 | 0.326 |  | 1.53 | 0.97;2.45 | 0.073 |  | 1.21 | 0.94;1.56 | 0.149 | |
| HSCT^f^ | 1.10 | 0.50;2.78 | 0.827 |  | 1.56 | 0.65;4.08 | 0.333 |  | 1.02 | 0.61;1.79 | 0.931 | |
| Year of study^g^ |  |  |  |  |  |  |  |  |  |  |  | |
| 2010-2013 |  |  |  |  | 0.44 | 0.28;0.68 | **<0.001** |  | 0.84 | 0.61;1.16 | 0.288 | |
| 2015-2017 | 0.96 | 0.58;1.63 | 0.892 |  |  |  |  |  |  |  |  | |
| 2021-2022 | 0.55 | 0.33;0.92 | **0.021** |  | 0.49 | 0.26;0.91 | **0.023** |  | 0.60 | 0.43;0.84 | **0.003** | |
|  | **Sunburn** | | | | | | | | | | | |
|  | **N = 1,013** | | | | **N = 542** | | | | **N = 1,837** | | | |
| Age at study | 1.12 | 1.05;1.19 | **<0.001** |  | 1.01 | 0.87;1.17 | 0.883 |  | 0.95 | 0.94;0.96 | **<0.001** | |
| Male^a^ | 0.98 | 0.73;1.34 | 0.920 |  | 0.72 | 0.51;1.02 | 0.067 |  | 1.04 | 0.86;1.26 | 0.697 | |
| Migration background^b^ | 0.54 | 0.35;0.82 | **0.005** |  | 0.71 | 0.44;1.15 | 0.172 |  | 0.46 | 0.35;0.62 | **<0.001** | |
| French/Italian language region^c^ | 0.93 | 0.66;1.28 | 0.647 |  | 1.13 | 0.76;1.66 | 0.551 |  | 0.87 | 0.70;1.09 | 0.221 | |
| Parental education^d^ |  |  |  |  |  |  |  |  |  |  |  |  |
| Secondary | 1.09 | 0.58;2.13 | 0.804 |  | 1.08 | 0.48;2.47 | 0.853 |  | 1.18 | 0.82;1.70 | 0.376 | |
| Tertiary | 0.90 | 0.49;1.76 | 0.759 |  | 1.33 | 0.58;3.14 | 0.504 |  | 1.49 | 1.03;2.17 | **0.037** | |
| Radiotherapy^e^ | 0.67 | 0.43;1.02 | 0.067 |  | 0.63 | 0.43;0.93 | **0.021** |  | 0.75 | 0.61;0.92 | **0.006** | |
| HSCT^f^ | 0.61 | 0.30;1.14 | 0.138 |  | 1.47 | 0.73;3.00 | 0.286 |  | 0.62 | 0.39;0.96 | **0.035** | |
| Year of study^g^ |  |  |  |  |  |  |  |  |  |  |  |  |
| 2010-2013 |  |  |  |  | 1.03 | 0.68;1.54 | 0.903 |  | 1.12 | 0.86;1.47 | 0.397 | |
| 2015-2017 | 1.57 | 1.07;2.31 | **0.021** |  | 1.05 | 0.58;1.87 | 0.879 |  | 1.34 | 0.99;1.82 | 0.060 | |
| 2021-2022 | 3.73 | 2.57;5.47 | **<0.001** |  | 1.80 | 1.00;3.31 | 0.052 |  | 2.57 | 1.89;3.52 | **<0.001** | |
|  | **Physician skin examinations** | | | | | | | | | | | |
|  | **N = 275^i^** | | | | **N = 538** | | | | **N = 1,807** | | | |
| Age at study | 0.86 | 0.73;1.00 | 0.057 |  | 1.02 | 0.83;1.26 | 0.841 |  | 1.02 | 1.00;1.04 | **0.020** | |
| Male^a^ | 0.98 | 0.46;2.11 | 0.958 |  | 1.38 | 0.85;2.27 | 0.199 |  | 0.65 | 0.51;0.84 | **0.001** | |
| Migration background^b^ | 0.83 | 0.32;1.98 | 0.690 |  | 1.27 | 0.66;2.34 | 0.451 |  | 1.38 | 0.98;1.94 | 0.064 | |
| French/Italian language region^c^ | 2.40 | 1.09;5.32 | **0.029** |  | 1.12 | 0.65;1.89 | 0.674 |  | 1.38 | 1.04;1.81 | 0.022 | |
| Parental education^g^ |  |  |  |  |  |  |  |  |  |  |  |  |
| Secondary |  |  |  |  | 1.50 | 0.50;5.66 | 0.503 |  | 0.96 | 0.62;1.50 | 0.848 | |
| Tertiary | 3.55 | 1.32;11.61 | **0.020** |  | 1.53 | 0.49;5.92 | 0.497 |  | 1.27 | 0.81;2.01 | 0.306 | |
| Radiotherapy^e^ | 2.42 | 0.87;6.27 | 0.076 |  | 1.14 | 0.67;1.91 | 0.620 |  | 1.02 | 0.78;1.33 | 0.880 | |
| HSCT^f^ | 3.82 | 1.42;9.93 | **0.006** |  | 2.09 | 0.88;4.63 | 0.078 |  | 2.05 | 1.27;3.23 | **0.003** | |
| Year of study |  |  |  |  |  |  |  |  |  |  |  |  |
| 2010-2013 |  |  |  |  | 0.82 | 0.46;1.46 | 0.511 |  | 1.31 | 0.93;1.83 | 0.123 | |
| 2015-2017 |  |  |  |  | 0.86 | 0.36;1.91 | 0.721 |  | 1.23 | 0.82;1.81 | 0.303 | |
| 2021-2022 |  |  |  |  | 1.05 | 0.46;2.24 | 0.909 |  | 1.03 | 0.67;1.54 | 0.887 | |

^a^ reference: female, ^b^ reference: no migration background, ^c^ reference: German language region, ^d^ reference: primary education, ^e^ reference: no radiotherapy; ^f^ reference: no HSCT, ^g^ year of study reference category for children is 2007-2013 and for adolescents and adults 2007 to 2009; ^h^ due to the lower n in this analysis, we combined primary and secondary education into in category for children; for adolescents and adults, we kept it separate; ^i^ Since we only asked preventive skin examinations for children in 2021/2022, we did not include it study year as exposure for preventive skin examinations in this age group, HSCT = hematopoietic stem cell transplantation

# **Supplementary Table 10.** Sensitivity analysis: Factors associated with sun protection and sunburn in multivariable logistic regression in child, adolescents, and adult childhood cancer survivors, including season

|  | **Children (5-15 years)** | | | | **Adolescents (16-19 years)** | | | | **Adults (≥20 years)** | | |
| --- | --- | --- | --- | --- | --- | --- | --- | --- | --- | --- | --- |
|  | *OR* | *95%CI* | *p* |  | *OR* | *95%CI* | *p* |  | *OR* | *95%CI* | *p* |
|  | **Sun protection** | | | | | | | | | | |
|  | **N = 1,013** | | | | **N = 476** | | | | **N = 1,626** | | |
| Age at study | 0.74 | 0.66;0.82 | **<0.001** |  | 0.94 | 0.79;1.12 | 0.496 |  | 1.00 | 0.97;1.04 | 0.763 |
| Male^a^ | 0.72 | 0.47;1.10 | 0.132 |  | 0.40 | 0.26;0.60 | **<0.001** |  | 0.51 | 0.40;0.65 | **<0.001** |
| Migration background^b^ | 0.69 | 0.42;1.17 | 0.162 |  | 0.57 | 0.34;0.97 | **0.038** |  | 0.58 | 0.43;0.80 | **0.001** |
| French/Italian language region^c^ | 1.30 | 0.82;2.09 | 0.274 |  | 1.12 | 0.70;1.81 | 0.644 |  | 1.05 | 0.80;1.39 | 0.707 |
| Parental education^d^ |  |  |  |  |  |  |  |  |  |  |  |
| Secondary | 2.01 | 0.98;4.00 | 0.051 |  | 1.42 | 0.60;3.33 | 0.417 |  | 1.31 | 0.88;1.95 | 0.181 |
| Tertiary | 1.97 | 0.98;3.84 | **0.050** |  | 1.92 | 0.78;4.73 | 0.156 |  | 1.56 | 1.02;2.37 | **0.040** |
| Radiotherapy^e^ | 1.32 | 0.76;2.42 | 0.344 |  | 1.62 | 1.02;2.59 | **0.043** |  | 1.20 | 0.93;1.55 | 0.169 |
| HSCT^f^ | 1.08 | 0.49;2.76 | 0.860 |  | 1.68 | 0.70;4.38 | 0.264 |  | 1.01 | 0.60;1.76 | 0.975 |
| Birth year | 0.95 | 0.90;1.00 | 0.074 |  | 0.99 | 0.93;1.05 | 0.738 |  | 0.96 | 0.94;0.99 | **0.002** |
| Season^g^ |  |  |  |  |  |  |  |  |  |  |  |
| Spring | 0.67 | 0.37;1.19 | 0.178 |  | 0.98 | 0.52;1.83 | 0.946 |  | 1.25 | 0.87;1.78 | 0.234 |
| Summer | 0.38 | 0.18;0.82 | **0.012** |  | 2.40 | 1.35;4.28 | **0.003** |  | 1.04 | 0.73;1.48 | 0.832 |
| Autumn | 0.88 | 0.45;1.72 | 0.713 |  | 1.38 | 0.74;2.57 | 0.313 |  | 0.84 | 0.54;1.29 | 0.415 |
|  | **Sunburn** | | | | | | | | | | |
|  | **N = 1,013** | | | | **N = 542** | | | | **N = 1,837** | | |
| Age at study | 1.28 | 1.19;1.38 | **<0.001** |  | 1.05 | 0.91;1.22 | 0.515 |  | 1.02 | 0.99;1.04 | 0.146 |
| Male^a^ | 0.98 | 0.73;1.33 | 0.917 |  | 0.73 | 0.52;1.04 | 0.084 |  | 1.07 | 0.88;1.29 | 0.522 |
| Migration background^b^ | 0.54 | 0.34;0.82 | **0.005** |  | 0.72 | 0.44;1.16 | 0.179 |  | 0.47 | 0.35;0.62 | **<0.001** |
| French/Italian language region^c^ | 0.95 | 0.67;1.32 | 0.744 |  | 1.09 | 0.73;1.62 | 0.671 |  | 0.87 | 0.70;1.08 | 0.209 |
| Parental education^d^ |  |  |  |  |  |  |  |  |  |  |  |
| Secondary | 1.09 | 0.58;2.14 | 0.791 |  | 1.10 | 0.49;2.51 | 0.823 |  | 1.15 | 0.80;1.66 | 0.454 |
| Tertiary | 0.90 | 0.49;1.75 | 0.756 |  | 1.33 | 0.58;3.12 | 0.504 |  | 1.42 | 0.98;2.07 | 0.067 |
| Radiotherapy^e^ | 0.67 | 0.43;1.02 | 0.067 |  | 0.65 | 0.44;0.95 | **0.028** |  | 0.76 | 0.62;0.94 | **0.010** |
| HSCT^f^ | 0.61 | 0.30;1.14 | 0.140 |  | 1.46 | 0.72;2.99 | 0.290 |  | 0.62 | 0.40;0.96 | **0.036** |
| Birth year | 1.14 | 1.10;1.20 | **<0.001** |  | 1.04 | 0.99;1.09 | 0.113 |  | 1.08 | 1.05;1.10 | **<0.001** |
| Season^g^ |  |  |  |  |  |  |  |  |  |  |  |
| Spring | 0.96 | 0.63;1.46 | 0.843 |  | 1.10 | 0.63;1.92 | 0.741 |  | 1.18 | 0.87;1.61 | 0.298 |
| Summer | 0.85 | 0.43;1.61 | 0.629 |  | 1.17 | 0.70;1.94 | 0.552 |  | 1.50 | 1.11;2.03 | **0.008** |
| Autumn | 0.90 | 0.57;1.42 | 0.655 |  | 0.84 | 0.50;1.40 | 0.499 |  | 1.15 | 0.83;1.60 | 0.408 |

^a^ reference: female, ^b^ reference: no migration background, ^c^ reference: German language region, ^d^ reference: primary education, ^e^ reference: no radiotherapy; ^f^ reference: no HSCT, ^g^ reference: winter season

# **Supplementary Table 11.** Sensitivity analysis: Factors associated with sun protection, sunburn and physician skin examination in multivariable logistic regression in adult childhood cancer survivors, including their own highest education obtained at the time of study

|  | **Sun protection** | | | | **Sunburn** | | | | **Physician skin examination** | | |
| --- | --- | --- | --- | --- | --- | --- | --- | --- | --- | --- | --- |
|  | *OR* | *95%CI* | *p* |  | *OR* | *95%CI* | *p* |  | *OR* | *95%CI* | *p* |
|  |  | | | | | | | | | | |
|  | **N = 1,603** | | | | **N = 1,812** | | | | **N = 1,783** | | |
| Age at study | 1.00 | 0.97;1.04 | 0.790 |  | 1.01 | 0.98;1.03 | 0.587 |  | 1.04 | 1.01;1.08 | **0.010** |
| Male^a^ | 0.50 | 0.39;0.64 | **<0.001** |  | 1.06 | 0.87;1.29 | 0.572 |  | 0.65 | 0.50;0.84 | **0.001** |
| Migration background^b^ | 0.57 | 0.42;0.77 | **<0.001** |  | 0.48 | 0.36;0.64 | **<0.001** |  | 1.37 | 0.96;1.92 | 0.075 |
| French/Italian language region^c^ | 1.00 | 0.76;1.31 | 0.987 |  | 0.84 | 0.67;1.04 | 0.110 |  | 1.41 | 1.07;1.85 | **0.014** |
| Parental education^d^ |  |  |  |  |  |  |  |  |  |  |  |
| Secondary | 1.24 | 0.82;1.86 | 0.300 |  | 1.07 | 0.74;1.55 | 0.725 |  | 0.99 | 0.64;1.57 | 0.953 |
| Tertiary | 1.44 | 0.93;2.22 | 0.101 |  | 1.31 | 0.89;1.92 | 0.172 |  | 1.29 | 0.81;2.08 | 0.290 |
| Radiotherapy^e^ | 1.22 | 0.94;1.58 | 0.137 |  | 0.78 | 0.64;0.96 | **0.020** |  | 0.98 | 0.74;1.28 | 0.865 |
| HSCT^f^ | 0.98 | 0.58;1.71 | 0.930 |  | 0.62 | 0.39;0.96 | **0.037** |  | 2.06 | 1.27;3.27 | **0.003** |
| Birth year | 0.97 | 0.94;0.99 | **0.005** |  | 1.07 | 1.05;1.09 | **<0.001** |  | 1.02 | 0.99;1.05 | 0.218 |
| Survivor’s education^g^ |  |  |  |  |  |  |  |  |  |  |  |
| Secondary | 1.10 | 0.72;1.65 | 0.646 |  | 1.92 | 1.34;2.77 | **<0.001** |  | 0.95 | 0.60;1.55 | 0.825 |
| Tertiary | 1.32 | 0.81;2.11 | 0.259 |  | 1.86 | 1.25;2.80 | **0.003** |  | 0.94 | 0.57;1.61 | 0.824 |

^a^ reference: female, ^b^ reference: no migration background, ^c^ reference: German language region, ^d^ reference: primary education, ^e^ reference: no radiotherapy; ^f^ reference: no HSCT, ^g^ reference: primary education. Survivor’s education represents the highest educational degree obtained at the time of study.
